# Gender Differences in Time Spent on Documentation and the Electronic Health Record in a Large Ambulatory Network

**DOI:** 10.1001/jamanetworkopen.2022.3935

**Published:** 2022-03-24

**Authors:** Lisa S. Rotenstein, Allan S. Fong, Molly Moore Jeffery, Christine A. Sinsky, Richard Goldstein, Brian Williams, Edward R. Melnick

**Affiliations:** 1 Department of Medicine, Brigham and Women’s Hospital, Boston, Massachusetts; 2 Harvard Medical School, Boston, Massachusetts; 3 MedStar Institute for Biomedical Informatics, Washington, District of Columbia; 4 Division of Health Care Delivery Research, Mayo Clinic, Rochester, Minnesota; 5 Division of Emergency Medicine, Mayo Clinic, Rochester, Minnesota; 6 American Medical Association, Chicago, Illinois; 7 Northeast Medical Group, Stratford, Connecticut; 8 Department of Emergency Medicine, Yale University School of Medicine, New Haven, Connecticut; 9 Department of Biostatistics (Health Informatics), Yale School of Public Health, New Haven, Connecticut

## Abstract

This cross-sectional study assesses gender differences in time spent on documentation and electronic health records in a large ambulatory care network.

## Introduction

Electronic health records (EHRs) have transformed physician work and work experiences. Although there are known positive effects of EHRs on clinical outcomes,^1^ EHRs have also been associated with clinician burnout.^2^ Prior work has demonstrated higher rates of burnout among women physicians.^3^ We sought to characterize gender differences associated with time spent on the EHR and the specific EHR activities associated with these differences.

## Methods

This cross-sectional study was deemed exempt from review and informed consent by the Yale University institutional review board because it used deidentified data and did not involve human participants. This study followed the Strengthening the Reporting of Observational Studies in Epidemiology (STROBE) reporting guideline.

This was a retrospective cross-sectional study of EHR use in a large, New England ambulatory practice network. Data for EHR use by nontrainee, ambulatory physicians between February 2018 and December 2019 were retrieved from Signal (Epic Systems). Scheduling data were derived from Clarity (Epic Systems), and demographic data were obtained from human resources records. Mean daily total time spent on the EHR (EHR-Time_8_), time spent on the EHR outside scheduled hours (work outside of work [WOW_8_]), and time spent on clinical documentation (Note-Time_8_) were retrieved and normalized to 8 hours of scheduled patient time, as previously described.^4^ We additionally retrieved information on mean daily in-basket time (IB-Time_8_) and time on orders (Ord-Time_8_) normalized to 8 hours, and percentage of orders with team member contribution (TW_ORD_).

Bivariate comparisons between demographic, productivity (including monthly patient volume, demand [percentage of appointments scheduled], intensity [patients per hour]), and EHR use metrics (EHR-Time_8_, WOW_8,_ Note-Time_8,_ IB-Time_8_ and Ord-Time_8_) for women vs men physicians were conducted using χ^2^ for categorical variables and Kruskal-Wallis tests for continuous variables. Generalized estimating equations (Poisson family, log link) with robust SEs grouped by physician were used to characterize adjusted gender differences in EHR-Time_8_, WOW_8,_ and Note-Time_8_. Models adjusted for specialty, age range, date range (enabling adjustment for potential seasonality), monthly patient volume, intensity (the quantity of both may influence physician documentation patterns), and TW_ORD_ (with team contribution to EHR functions potentially influencing EHR and note time). Analyses were performed with Python software version 3.7 (Python Software Foundation). *P* values were 2-sided, and significance was assessed at α = .05.

## Results

Complete data were available for 318 physicians (95% of potential sample); 194 physicians (61.0%) were men and 124 physicians (39.0%) were women, and 231 physicians (72.6%) were aged 45 years or older. More than half the sample was primary care physicians (173 physicians [54.4%]); 103 physicians (32.3%) were medical specialists, and 42 physicians (13.2%) were surgical specialists (Table). Women physicians were younger, more represented in primary care specialties, and cared for significantly fewer patients per hour and month (Table).

**Table.** Demographic Characteristics, Productivity, and EHR Use by Physician Gender

Characteristic	Physicians, No. (%)			*P* value
	All (N = 318)	Female (n = 124)	Male (n = 194)	
Age, y				
<35	22 (6.9)	12 (9.7)	10 (5.2)	<.001^a^
35-44	65 (20.4)	36 (29.0)	29 (14.9)	
45-54	100 (31.4)	50 (40.3)	50 (25.8)	
55-64	79 (24.8)	22 (17.7)	57 (29.4)	
≥65	52 (16.4)	4 (3.2)	48 (24.7)	
Specialty type				
Medical specialty	103 (32.3)	25 (20.2)	78 (40.2)	<.001^a^
Primary care	173 (54.4)	82 (66.1)	91 (46.9)	
Surgical specialty	42 (13.2)	17 (13.7)	25 (12.9)	
Months in analysis per physician, mean (SD)	18.4 (5.9)	18.5 (5.8)	18.3 (6.0)	.26^b^
Productivity, mean (SD), No.				
Monthly patient volume	214 (91)	185 (72)	216 (87)	<.001^b^
Demand	74 (18)	73 (19)	72 (18)	.22^b^
Intensity	2.6 (0.8)	2.2 (0.7)	2.7 (0.8)	<.001^b^
EHR Use, mean (SD), h				
EHR-Time_8_	5.4 (1.8)	5.9 (1.8)	5.3 (1.8)	<.001^b^
WOW_8_	0.8 (0.7)	0.9 (0.7)	0.8 (0.7)	.02^b^
Note-Time_8_	1.8 (0.9)	2.1 (0.9)	1.7 (0.9)	<.001^b^
IB-Time_8_	0.7 (0.4)	0.7 (0.4)	0.7 (0.4)	.36^b^
Ord-Time_8_	0.8 (0.4)	0.9 (0.3)	0.8 (0.4)	.17^b^
TW_ORD_ (%)	23 (21)	22 (19)	23 (22)	.49^b^

Abbreviations: EHR, electronic health record; EHR-Time_8_, mean daily total time spent on the EHR; IB-Time_8_, mean daily in-basket time; Note-Time_8_, time spent on clinical documentation; Ord-Time_8_, time on orders; TW_ORD_, orders with team member contribution; WOW_8_, work outside of work.

^a^ Comparisons made via χ^2^ tests.

^b^ Comparisons made via Kruskal-Wallis tests.

In unadjusted analyses, women physicians, compared with men, had higher EHR-Time_8_ (mean [SD], 5.9 [1.8] hours vs 5.3 [1.8] hours; *P* < .001), WOW_8_ (mean [SD], 0.9 [0.7] hours vs 0.8 [0.7] hours; *P* = .02), and Note-Time_8_ (mean [SD]. 2.1 [0.9] hours vs 1.7 [0.9] hours; *P* < .001) (Table). Gender differences persisted in multivariable analyses (Figure). In adjusted analyses, women physicians spent a mean of 41.4 (95% CI, 18.4-63.8) minutes more in EHR-Time_8_ than men (*P* < .001). Mean adjusted WOW_8_ was 9.6 (95% CI, 9.1-10.1) minutes greater for women vs men physicians (*P* = .04), while mean adjusted Note-Time_8_ was 31.0 (95% CI, 15.4-49.5) minutes greater for women physicians (*P* < .001). This translated to the following mean (SD) adjusted times for women vs men: EHR-Time_8_ were 5.81 (1.79) hours vs 5.23 (1.83) hours, WOW_8_ were 0.91 (0.74) hours vs 0.75 (0.70) hours, and Note-Time_8_ was 2.03 (0.90) hours vs 1.67 (0.87) hours.

**Figure.**
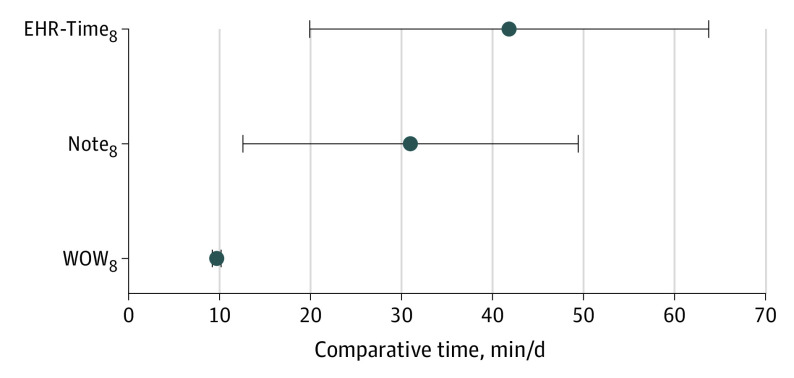
Comparisons of Additional Adjusted Mean Daily Time Spent on the Electronic Health Record (EHR-Time_8_), Time Spent on EHR Outside Scheduled Hours (WOW_8_), and Time Spent on Clinical Documentation (Note-Time_8_) for Women vs Men Physicians Values are calculated per 8 hours of scheduled patient time.

## Discussion

In this cross-sectional study across ambulatory specialties, we demonstrated that female physicians spend more time on the EHR overall, after-hours, and on EHR-based documentation than male physicians. Clinical documentation is the primary activity driving gender differences in EHR time. These differences persisted after accounting for hours worked, physician specialty, and other characteristics, despite female physicians caring for slightly fewer patients on average.

Our findings provide a potential mechanism for the gender gap in burnout,^3^ which has implications for workforce mental health^5^ and physician retention.^6^ They suggest that women physicians may benefit from policy changes, workflows, and technologies that reduce documentation burden, including scribes, team documentation, and artificial intelligence–powered solutions.

Strengths of our study include the examination of EHR use patterns from a large, multispecialty ambulatory care network and the availability of detailed demographic, productivity, and EHR use information. Limitations include derivation of data from a single, nonteaching practice network and availability of data only from prior to December 2019. Future studies should characterize workflow and technology interventions that can reduce time spent on the EHR, with focus on interventions that reduce documentation burden.

## References

[1] Lin SC, Jha AK, Adler-Milstein J. Electronic health records associated with lower hospital mortality after systems have time to mature. Health Aff (Millwood). 2018;37(7):1128-1135. doi:10.1377/hlthaff.2017.1658 29985687

[2] Adler-Milstein J, Zhao W, Willard-Grace R, Knox M, Grumbach K. Electronic health records and burnout: time spent on the electronic health record after hours and message volume associated with exhaustion but not with cynicism among primary care clinicians. J Am Med Inform Assoc. 2020;27(4):531-538. doi:10.1093/jamia/ocz220 32016375 PMC7647261

[3] Marshall AL, Dyrbye LN, Shanafelt TD, . Disparities in burnout and satisfaction with work-life integration in U.S. physicians by gender and practice setting. Acad Med. 2020;95(9):1435-1443. doi:10.1097/ACM.0000000000003521 32459677

[4] Melnick ER, Ong SY, Fong A, . Characterizing physician EHR use with vendor derived data: a feasibility study and cross-sectional analysis. J Am Med Inform Assoc. 2021;28(7):1383-1392. doi:10.1093/jamia/ocab011 33822970 PMC8279798

[5] Rotenstein LS, Zhao Z, Mata DA, Guille C, Sen S. Substantial overlap between factors predicting symptoms of depression and burnout among medical interns. J Gen Intern Med. 2021;36(1):240-242. doi:10.1007/s11606-020-05664-x 32026254 PMC7859012

[6] Shanafelt TD, Mungo M, Schmitgen J, . Longitudinal study evaluating the association between physician burnout and changes in professional work effort. Mayo Clin Proc. 2016;91(4):422-431. doi:10.1016/j.mayocp.2016.02.001 27046522

